# Monitoring and Evaluating Progress towards Universal Health Coverage in India

**DOI:** 10.1371/journal.pmed.1001697

**Published:** 2014-09-22

**Authors:** Narayanan Devadasan, Soumitra Ghosh, Sunil Nandraj, T. Sundararaman

**Affiliations:** 1 Institute of Public Health, Bengaluru, India; 2 Tata Institute of Social Sciences, Mumbai, India; 3 Public Health Foundation of India, New Delhi, India; 4 National Health Systems Resource Centre, New Delhi, India

## Abstract

This paper is a country case study for the Universal Health Coverage Collection, organized by WHO. N. Devadasan and colleagues illustrate progress towards UHC and its monitoring and evaluation in India.

*Please see later in the article for the Editors' Summary*

This paper is part of the PLOS Universal Health Coverage Collection. This is the summary of the India country case study. The full paper is available as Supporting Information file Text S1.

## Background

Universal Health Coverage (UHC) is gaining in importance across the world. WHO defines UHC as “ensuring that all people can use the promotive, preventive, curative, rehabilitative and palliative health services they need, of sufficient quality to be effective, while also ensuring that the use of these services does not expose the user to financial hardship” [1]. While many countries have achieved UHC to a large extent, others are still on the path; India being one such country.

## Universal Health Coverage: The Policy Context in India

UHC is not a new concept in India. The Bhore committee (1946) had recommended that India should have a health system that “is designed to provide [a full range of health care] for everyone who wishes to use it. ….everyone who uses the new service is assured of ready access to whichever of its branches he or she needs” [2]. However, since then the government has steadily diluted its promise of universal health care. In 1983, it only promised access to “universal provision of comprehensive primary health care services” [3] and in 2002, UHC became reduced to access to “universal immunisation services” [4]. The government's flagship programme National Rural Health Mission (NRHM), launched in 2005, did include strengthening health services, but there was no mention of universal provision of health services [5]. It was only in 2011, when the government of India commissioned a High Level Expert Group (HLEG) that UHC once again entered the lexicon of health policy makers. The HLEG report recommended that India should achieve UHC by 2022 by increasing government finances and by strengthening provision of government health services [6]. This report was accepted by the Planning Commission and is the focus of the health chapter of its 12th five-year plan [7].

## Monitoring and Evaluation for UHC

The Ministry of Health (MoH) has a digitised health management information system (HMIS) that collects data every month from all the government health facilities across the country. However, the HMIS has a major weakness; it does not collect information from the private health sector. Since the private sector is the main provider of many health services in India, the HMIS reports are incomplete. Thus policy makers and managers are unable to determine the true health status of the citizens of the country.

To understand the extent to which people are covered by various health services, the policy maker then relies on periodic household surveys administered by independent agencies [8],[9]. Outpatient and inpatient services and health expenditure are captured through the decennial morbidity surveys [10]. Infant mortality rates and occasionally the maternal mortality ratio can be obtained through annual Sample Registration Surveys [11]. Some states have recently started an annual health survey [12].

## Progress towards UHC in India

Given the paucity of routine and disaggregated data, it is difficult to assess the status of UHC in India. We used data from household surveys done in 2004, 2005, and 2009 to calculate the population, service, and financial coverage. We selected indicators on the basis of their availability across these surveys. In 2009, only 53% of pregnant women had received three check-ups and only 61% of infants were fully immunised. Disaggregating by socio-economic parameters, shows that only 27% of pregnant women belonging to the poorest quintile had received a full antenatal check-up. Nearly 50% of all women who had an antenatal check-up had to make direct out-of-pocket (OOP) payments for their examination.

**Figure 1:**
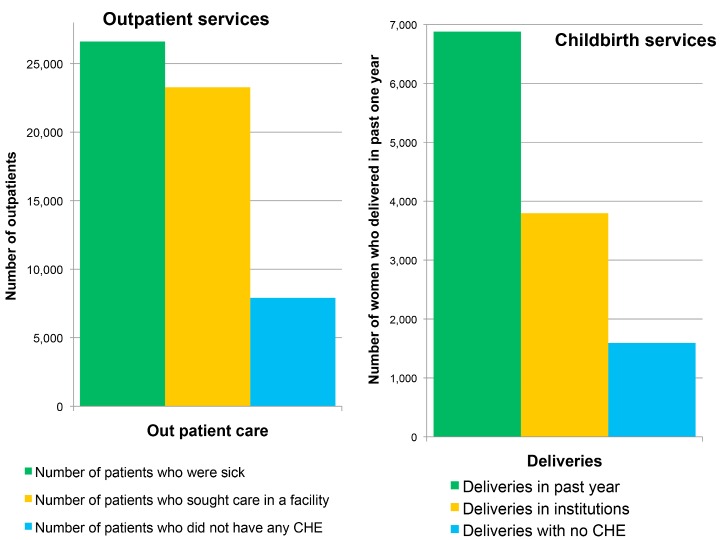
Service and financial coverage for outpatient and natal services in India (2004). Source: NSS 60th round, 2004 [10].

Among patients who fell ill, more than 80% sought care at a formal health facility. Similarly more than 70% of women had delivered at a health facility in 2009. However, among the poorest quintile this figure was only 55%. Also, most of the patients or women had to make OOP payments to receive these services (Figure 1).

Only 47% of households in India had access to drinking water within their premises. The rest of the households had to walk distances ranging from 100 metres to a few kilometres to get water.

## Conclusions and Recommendations

**Figure 2:**
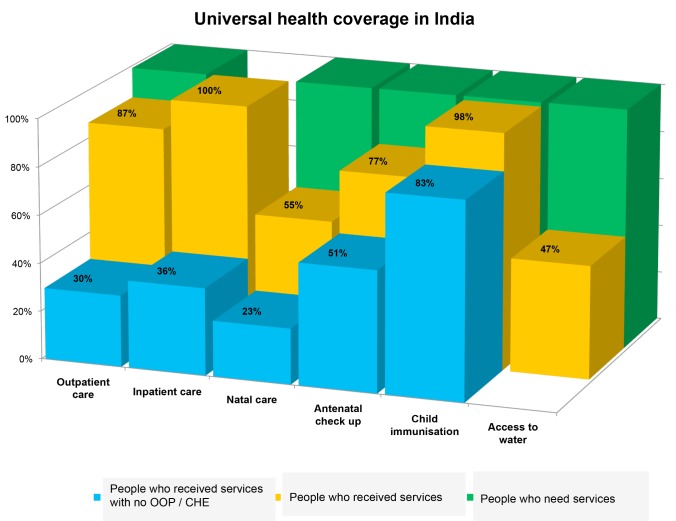
Universal health coverage for selected health services in India in 2004. Source: NFHS 3, DLHS 3, NSS 60th round, and Coverage evaluation Survey [8]–[10],[15].

India has pledged to achieve UHC by 2022. We found that while the population is reasonably covered by preventive and curative health services, financial coverage is lacking for most of these services (Figure 2). When we disaggregate coverage by population subgroups, we consistently find that people living in rural areas, belonging to lower caste households, and having a low economic status use the services much less compared to other groups.

For India to move towards UHC, the first step should be to provide financial protection against medical expenses. Although there are currently many subsidised health insurance schemes for poor people [13], they do not address the main source of OOP payments, which is ambulatory care and medicines [14]. One of the measures that the government of India could take to improve coverage would be to provide access to free medicines for all individuals seeking care, which would reduce OOP payments considerably. The second measure would be to extend existing services to the most vulnerable sections of the population, that is the poorest, aboriginals, and others. With these two measures in place, the government could undertake additional actions to attain UHC. Another important stage would be to monitor progress against specific milestones. Because the current HMIS is inadequate, the government must employ periodic household surveys to capture the necessary information.

## Supporting Information

Text S1 **The full country case study for India.**
(DOCX)

## Abbreviations

HMIS: health management information system
OOP: out-of-pocket
UHC: universal health coverage
