# Supplementary material for: Monitoring and Evaluating Progress towards Universal Health Coverage in India
Source: PLoS Med. 2014 Sep 22;11(9):e1001697. doi: 10.1371/journal.pmed.1001697 (PMC4171464; doi:10.1371/journal.pmed.1001697)
Supplement: Text S1 — The full country case study for India. (DOCX) [file pmed.1001697.s001.docx]

**Full Case Study: Monitoring and evaluating progress towards Universal Health Coverage in India**

Narayanan Devadasan^1*^, Soumitra Ghosh^2^, Sunil Nandraj^3^ and Thiagrajan Sundararaman^4^

^1^ Institute of Public Health, Bangalore, India

^2^ Tata Institute of Social Sciences, Mumbai, India

^3^Public Health Foundation of India, New Delhi, India

^4^National Health Systems Resource Centre, New Delhi, India

*Corresponding author: Narayanan Devadasan

Email: deva@iphindia.org

**This paper is the full country case study to accompany the summary paper “Monitoring and evaluating progress towards Universal Health Coverage in India” that is part of the Universal Health Coverage Collection. Not commissioned; externally reviewed.**

**Abstract:** Universal health coverage (UHC) is being promoted as the main agenda, post 2015. While UHC is not new to India, it was only in 2011 that the government appointed a high level expert group to develop a strategy to achieve UHC. Many of the recommendations of this report have been incorporated into the country’s 12^th^ five year plan (2012 to 2017).

Unfortunately, currently, it is not clear where India is on the road towards UHC. Routine data that is collected has significant deficiencies and cannot be relied upon. This then leaves us with datasets from periodic household surveys to calculate the extent of population, services and financial coverage.

Using such datasets from different surveys, we have estimated service coverage (preventive and curative care); population coverage (lower castes, women and rural / urban populations) and financial coverage (Out of pocket payments).

We find that in 2009, only 53% of pregnant women had received three antenatal check-ups and only 61% of infants were fully immunised. Disaggregating this by socio-economic parameters, one finds that only 27% of pregnant women belonging to the poorest quintile had received full antennal check-up. Nearly 50% of all women who had an antenatal check-up had to make direct out-of-pocket (OOP) payments for their examination.

Among patients who fell ill, more than 80% sought care at a formal health facility. Similarly more than 70% of pregnant women had delivered at a health facility in 2009. However, among the poorest quintile this figure was only 55%. Also, most of the patients or women had to make OOP payments to receive these services.

India has pledged to achieve universal health coverage (UHC) by 2022. We found that while the population is reasonably covered by preventive and curative health services, financial coverage is lacking for most of these services.

**Summary Points:**

1. India has committed to moving towards universal health coverage through a tax based financing mechanism.
2. A wide range of health services are provided by both the government and private health sector. Unfortunately, the government services are inadequate, so those patients who can afford it, seek care from the private sector. This results in high out-of-pocket payments.
3. Using existing and nationally representative surveys, we measure the level to which India has achieved UHC.
4. We find that while service coverage is good, there are gaps in population coverage, especially for the socially and economically vulnerable groups.
5. Nearly all patient groups are exposed to high out-of-pocket payments and are inadequately protected from financial catastrophe.
6. The government needs to address these two gaps as soon as possible and also need to monitor the progress towards UHC through regular and periodic surveys that capture all the dimensions of UHC.

**1. Background**

The concept of universal health coverage (UHC) is gaining in importance across the world. First enunciated in the World Health Report of 2000 [1], it gained legitimacy when the World Health Assembly passed a resolution to this effect in 2005 [2]. The theme in the World Health reports of 2010 and 2013 [3, 4] was also on UHC. Margaret Chan, the Director-General of the World Health Organization (WHO) calls UHC “the single most powerful concept that public health has to offer” [5]. WHO defines UHC as “ensuring that all people can use the promotive, preventive, curative, rehabilitative and palliative health services they need, of sufficient quality to be effective, while also ensuring that the use of these services does not expose the user to financial hardship” [6]. It is important to note that we define coverage as “the ability [of a health service] to transform the intention to serve people into a successful intervention for their health” [7]. Tanahashi differentiates between potential coverage (the proportion of the target population who are eligible to receive the benefits from the health services) and the actual coverage (the proportion of eligible population who has received the necessary service). To give an example, citizens in countries that use tax based financing to finance and provide health services are ‘potentially covered’ by these health services. However, if some services are not uniformly available, then those who are able to utilise these services are the ones who are ‘actually covered’. In this document, we refer to coverage in terms of actual coverage.

Many countries have progressed far ahead in the path to UHC; others are in a relatively early stage on this path. Countries have used different financing mechanisms like taxes and insurance contributions to move towards UHC [8]. Most of the countries that are near the ideal of UHC are high income countries, though some low and middle income countries have made impressive strides on this path. While some countries have taken a long time to attain their current level of achievement [9], others have caught up in a couple of decades [10]. While there are a growing number of advocates for UHC, the evidence that UHC has an impact on health status of individuals and communities is equivocal. A probable reason for this could be the fact that UHC does not address many other social determinants of health [11].

| **Box S1: Indian health services**  India is a country of contrasts. On the one hand, it is home to some of the richest people in the world, and on the other hand, nearly half its children are undernourished. Patients from Europe, Africa and America seek care in India’s multi-speciality private hospitals, but the health status of Indians is worse than some of its immediate neighbours.  Health services in India are pluralistic and are provided by practitioners of modern medicine as well as that of Ayurveda, Unani, Homeopathy, Siddha and Yoga. The above are formally qualified practitioners, but there are enormous numbers of unqualified practitioners who practice with impunity as regulations are negligible.  The three tiered government health services provide the majority of preventive services as well as nearly half the secondary services. On the other hand, the private health services are the main providers for ambulatory care. The Indian health services are financed mainly through out-of-pocket (OOP) expenses by individual households at the point of care. |
| --- |

**2. Universal health coverage: the policy context**

The concept of UHC is not new to India. The Bhore committee had recommended, way back in 1946, that India should have a health system that “*is designed to provide [a* ***full range*** *of health care] for* ***everyone*** *who wishes to use it. ….everyone who uses the new service is assured of* ***ready access*** *to whichever of its branches he or she needs”* (emphasis added by author) [12]. These words are akin to the three dimensions of UHC: population, service and financial coverage as enunciated by the WHO [13]. To meet these goals, the government decided to both finance and provide health services, along the lines of the British National Health Services (NHS) [14,15]. The government set up a network of primary health centres and referral hospitals and employed doctors and nurses to provide the necessary health care. Along with this, various vertical health programmes were introduced to control communicable diseases like malaria, leprosy and tuberculosis. The focus was also on reproductive and child health programmes to provide health care for the vulnerable population. All these services were supposed to be free at the point of care [16,17].

However, these aspirations did not materialise, and it was clear that the government had failed in providing the necessary services. In the wake of the Alma Ata declaration, the government introduced its first health policy where it admitted that “*the demographic and health picture of the country still constitutes a cause for serious and urgent concern”* [18]. The government blamed this situation on the “Western, hospital centric model” and advocated for “*universal provision of comprehensive primary health care services*”. So from a full range of health services, the service coverage was reduced to primary health care services. At the turn of the millennium, India introduced its second health policy [19]. Over the two decades, between these two policies, the number of primary health centres had tripled, while the number of doctors had doubled. However, communicable diseases were still rampant, life expectancy had increased by a mere 10 percent points and infant mortality had reduced only by 30%. The term “universal” was now restricted to the immunisation programme. The policy blamed the chronic underfunding of the health sector by the government for this dismal performance.

With the election of a new socialist government in 2004, and with various studies and surveys highlighting the less than desirable health status of Indians [20,21] the government of India launched the National Rural Health Mission (NRHM) in 2005 [22]. This programme was expected to infuse more funds into the health sector, bring about architectural changes in this sector [23] and strengthen the process of decentralised planning. In 2006, a nationwide morbidity survey showed that despite 60 years of ‘free’ government health care services, most patients preferred to go to the private sector for curative care [24]. In the process, they incurred high out-of-pocket (OOP) payments that were impoverishing for over 60 million Indians [25]. In order to protect poor households from high OOP medical expenses, the government of India launched the National Health Insurance Scheme (RSBY) in 2008. All those below the poverty line (BPL) were eligible to enrol in the RSBY. In return for a token registration fee of INR 30 (US$ 0.5) per family per year, the family was covered against hospitalisation expenses up to US$ 500 per family per year [26]. The RSBY has enrolled over 37 million households as of March 2014 [27]. Recognising that a sum assured of US$ 500 would not be enough for treating conditions like cardiac ailments and cancers, four provincial governments also introduced an additional health insurance scheme to protect the poor against tertiary care expenses. These schemes again target the poor and have enrolled about 150 million individuals [28].

As a prelude to the 12^th^ five year plan, the government set up a High Level Expert Group (HLEG) to explore the possibility of achieving UHC in India. The HLEG report defined UHC as “*ensuring equitable access for all Indian citizens, resident in any part of the country, regardless of income level, social status, gender, caste or religion, to affordable, accountable, appropriate health services of assured quality (promotive, preventive, curative and rehabilitative) as well as public health services addressing the wider determinants of health delivered to individuals and populations with the government being the guarantor and enabler, although not necessarily the only provider, of health and related services*” [29]. Some of its main recommendations are provided in Box S2.

| **Box S2*:* Some key recommendations of the HLEG report**   - Increase government health expenditure to at least 3% of GDP by 2022 and spend this mostly on primary health care. - Develop a package of services for each of the five levels (community to district hospitals) and then provide these services either through the government facilities or by contracting-in these services from the private sector. - Reorient health care services to primary health care and also strengthen the district hospitals. - Ensure adherence to quality assurance standards - Increase Human Resources for Health density to achieve the WHO norms of 23 per 10,000 population, - Strengthen the role of civil society and community in the health services and introduce a grievance redressal mechanism - Ensure free availability of medicines in government facilities and also enforce price regulations for essential drugs. - Ensure the rational use of drugs - Introduce a public health cadre to strengthen the management of the health services - Introduce various regulatory authorities to improve governance of the health department. |
| --- |

The Planning Commission has accepted some of these recommendations in its 12^th^ five year plan [30]. It has agreed to “*work towards the long term objective of establishing a system of Universal Health Coverage*”, strengthen and expand the government health services, increase the government expenditure on health to at least 2.5% of GDP by 2017, contract in services from the private sector where necessary, increase the density of human resources and make available free generic medicines.

Thus the tentative scenario in 2014 is as follows; all Indians (1.25 billion of them) have potential access to ‘free’ government health services. Also since the state provides a variety of these free services, ranging from antenatal check-ups to cancer therapy; one should be able to declare India as having achieved UHC. However, due to chronic low government expenditure on health care, there is only one primary health centre (PHC) for 34,641 people, one government doctor for about 20,000 rural population [31] and most public health facilities do not have adequate medicines [32,33]. This forces patients to seek care in the private health sector where they have to make high OOP payments. Thus while all the citizens of India are ‘potentially covered’ by the government health services, the ‘actual coverage’ is insufficient. The Central Government Health Scheme (CGHS) and the Employees State Insurance Scheme (ESIS) provide comprehensive cover for the civil servants and industrial workers (59 million) respectively [28].Highly subsidised government health insurance schemes like RSBY and Vajpayee Arogyasree Scheme (VAS) assure approximately 200 million poor Indians of free hospital care. Some of the middle class (55 million) have purchased private health insurance to protect themselves against hospitalisation expenses. The rest of the population (nearly 900 million) have minimal health security and have to choose from an inadequately functioning public health service or an expensive private health service.

It is in this context that we try to assess the extent to which India is on the path to UHC. We identify the populations that are partially covered, the services that are not available and the extent to which the people have to pay for available services. Rather than conducting primary surveys, we review existing data sets to answer the above questions.

**3. Monitoring and evaluation for UHC**

To understand the status of UHC in India (or any country); one needs to look at indicators across the three domains of population coverage, service coverage and financial coverage. There is not yet a consensus about the ideal indicator for each of these domains. Various authors have used different indicators to measure UHC ranging from antenatal check-ups to median price of medicines [10,34,35]. Giedion, in a review of 41 studies on UHC has identified many of the indicators used to measure UHC (Table S1) [36]. However, the obstacle for most countries is the availability of reliable and valid data to calculate these indicators.

| **Indicator** | **Number of studies that have used this indicator** |
| --- | --- |
| Incidence and intensity of out-of-pocket expenditure | 13 |
| Outpatient contact rate | 12 |
| Mortality rates | 12 |
| Neonatal mortality rate | 3 |
| Infant mortality rate | 3 |
| Child mortality rate | 3 |
| Maternal mortality ratio | 2 |
| Standardised mortality rates | 1 |
| Self-assessment of health | 9 |
| Admission rate | 8 |
| Nutritional status | 8 |
| Incidence of low birth weight | 2 |
| Body Mass Index | 3 |
| Prevalence of anaemia | 1 |
| Child hood under nutrition | 2 |
| Incidence and intensity of catastrophic health expenditure | 6 |
| Utilisation of prenatal care | 4 |
| Access to safe delivery | 4 |
| Morbidity rate in the past month | 3 |
| Access to preventive care | 2 |
| Access to care for childhood diseases | 2 |
| Incidence and intensity of impoverishment | 2 |
| Prevalence of chronic illness | 2 |
| Complications after delivery | 2 |

Note: Indicators that were used in only one study (n=13) have not been included in this table.

**Table S1:** Common indicators used to measure Universal Health Coverage

Data source: Modified from Giedion et al (2013). A review of Universal Health Coverage in the Developing world: a review of existing evidence

The Indian health system has some of this information at the national and sub-national level, but most of them are from periodic household surveys rather than routine information systems (Table S2)

| Source of information | Indicator | Details of this study / survey | Remarks |
| --- | --- | --- | --- |
| Morbidity surveys by the National Sample Survey Organisation | Out-of-pocket expenses  Catastrophic health expenditure (CHE)  Impoverishment  Outpatient contact rate  Admission rate  Morbidity rates | 42^nd^ Round – 1986-87. A total of 50,770 households surveyed.  52^nd^ Round – 1998. A total of 120,942 households surveyed from 12,654 villages / urban units [37].  60^th^ Round – 2006. A total of 76, 868 households from 7,423 villages / urban units were surveyed [24]. | Two stage stratification was used to select the villages and households.  Survey conducted by the National Sample Survey Organisation, which is part of the Statistics division of the Ministry of Planning.  The only reliable source of outpatient and inpatient utilisation rates as well as out-of-pocket expenses in India. |
| Sample Registration System | Infant mortality rates | Latest round – 2013 [38]. | Annual sample surveys by the Ministry of Home Affairs. |
|  | Maternal mortality ratio | Latest round - 2011 [39] |  |
| National Family Health Surveys | Nutritional status  Maternal health indicators  Contraception indicators  Child health indicators | Round 1 – 1995. A total of 88,562 households were interviewed [40]  Round 2 – 2000. A total of 91,196 households were interviewed [21]  Round 3 – 2007. A total of 109,041 households were interviewed [41] | Two stage stratification was used.  Survey conducted by an independent organisation – the International Institute of Population Sciences, Mumbai, India.  It is a nationwide survey and gives figures disaggregated to the state level. |
| District Level Household & Facility Survey | Nutritional status  Maternal health indicators  Contraception indicators  Child health indicators  Government health facility indicators | Round 1 – 2000. A total of 529, 817 households were surveyed [42].  Round 2 – 2005. A total of 620, 107 households were interviewed [43].  Round 3 – 2009. A total of 684,267 households were interviewed. Also data about government health facilities at the district, sub-district and village level were collected [44]. | Multi stage stratified probability proportional to size sampling design was used. Currently married women were the respondents except in Round 3 when women between 19 and 49 were interviewed.  Survey conducted by an independent organisation – the International Institute of Population Sciences, Mumbai, India.  It is a nationwide survey and gives figures disaggregated to the district level.  The only survey that gives district level indicators. |

**Table S2:** Possible sources of information for measuring universal health coverage in India

Other than the above surveys, the Coverage Evaluation Survey (CES) provides the latest information about maternal and child health indicators [45] and the Census 2011 gives information about households [46]. The government of India also collects routine information through a monthly Health Management Information System (HMIS). Unfortunately, this HMIS is incomplete (as it does not capture private health sector data) and of poor quality [47,48].

We shortlisted the following indicators based on their usage in other studies and their availability in the nationally representative datasets. We were further restricted by the paucity of indicators for financial coverage and had to restrict our list to some maternal and child health indicators, as well as a few indicators for curative care.

**Preventive services**:

- Service coverage:
  - Proportion of pregnant women who received at least three antenatal check-ups
  - Proportion of post-natal women who received at least two postnatal check-ups
  - Proportion of eligible couples who use modern methods of contraception
  - Proportion of children (12 to 23 months) who are fully immunised
- Population coverage
  - Proportion of women who received at least three antenatal check-ups disaggregated by social, economic and geographic parameters. For geographic parameters, we have used indicators from rural and urban as well as from Tamil Nadu (a well performing region) and Odisha (a poorly performing region in 2005).
- Financial coverage
  - Proportion of women who made OOP payments for antenatal check-up
  - Proportion of families who made OOP payments for the vaccination of their child

**Curative services**

- Service coverage
  - Of the people who fell sick in the past 15 days, how many used outpatient services in a health facility
  - Proportion of children with acute respiratory infections (ARI) who received treatment in a health facility
  - Proportion of pregnant women who delivered in a facility
- Population coverage
  - All the above variables disaggregated by social, economic and geographic parameters
- Financial coverage
  - Proportion of outpatients who sought care and did not experience CHE.
  - Proportion of pregnant women who delivered in a facility and did not experience CHE.

**Promotive services**

- Service coverage
  - Proportion of families that have access to tapped drinking water in their house
  - Proportion of youth who are aware about the ill effects of tobacco

**Box S3**

We have considered a household to have experienced CHE if this household spent more than 40% of its capacity to pay. The capacity to pay was defined by the poverty lines drawn by the Planning commission. Any expenditure about this poverty limit was considered as non-subsistence spending and was used as a proxy for capacity to pay. Whenever household consumption expenditure was less than poverty limit, capacity to pay was defined as zero.

- In case of outpatient care, the reference period was 15 days. The OOP for outpatient care was converted into monthly figures and then CHE for outpatient care was calculated as health expenditure on outpatient care that exceeds 40% of household’s monthly capacity to pay.
- On the other hand, the reference period for IP was one year, and so the monthly household consumption expenditure was converted into annual household consumption expenditure for calculation of catastrophic payments.
- In the case of preventive services, we used the incidence of OOP payments rather than CHE.

Only direct health expenditure was used for calculating the above indicators.

**4. Progress towards UHC in India**

Preventive services

The main policy emphasis of the Indian health services has been a strong preventive health care programme. This focusses on the mother and the child, but also includes prevention and control of communicable and non-communicable diseases. In terms of coverage, government usually relies on periodic surveys to provide a true picture as routine information systems have uneven quality and data gaps across states. Using these surveys, we depict the coverage of some key services over time (Figure S3).

While there has been a varied but significant improvement in coverage of preventive services, with some states showing impressive gains and others still having a long way to catch up; the current level of achievement for most services in 2009 was in the range of 50% to 60% [45]. More worrying was that if one disaggregates antenatal care by specific socio-economic and geographic groups, then one finds that only 27% of women belonging to the poorest quintile have received more than three antenatal check-ups (Figure S4). Only 12% of women in this group have received full check-up (i.e. at least three check-ups plus TT vaccination plus 100 tablets of iron and folic acid). If one looks at the quality of antenatal check-ups, then one finds that the blood pressure was checked only for 75% of the pregnant women; only 27% of pregnant women were informed that bleeding per vagina is a sign of danger and only 48% of pregnant women received any contraceptive advice.


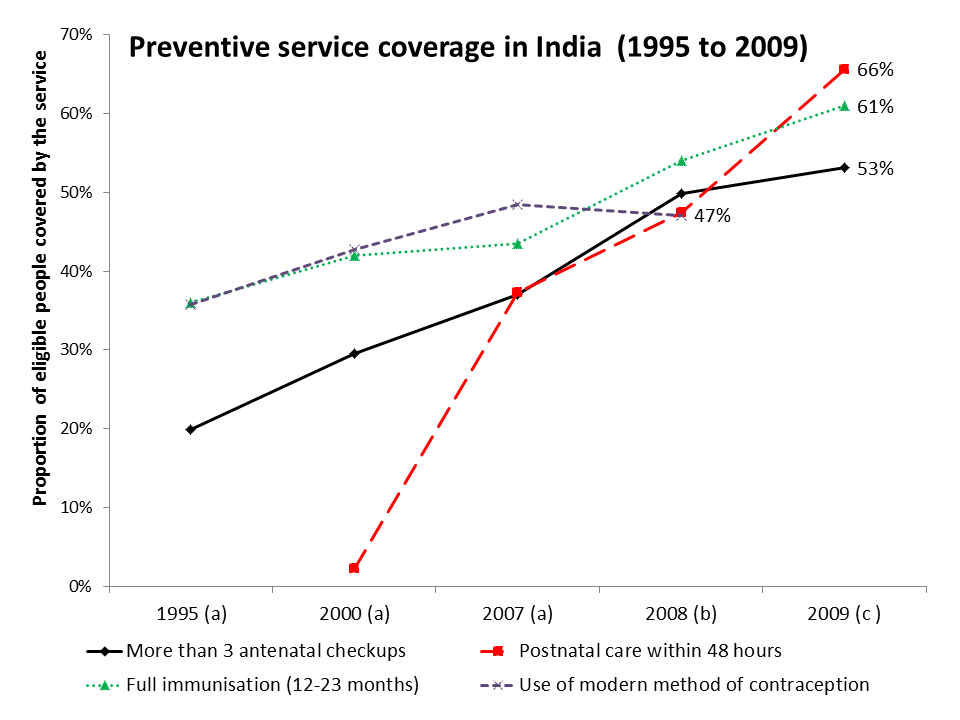


**Figure S3:** Coverage of select preventive health care services in India (1995 to 2009)

Data Sources: (a) National Family Health Survey (b) District Level Health Survey (c) Coverage Evaluation Survey


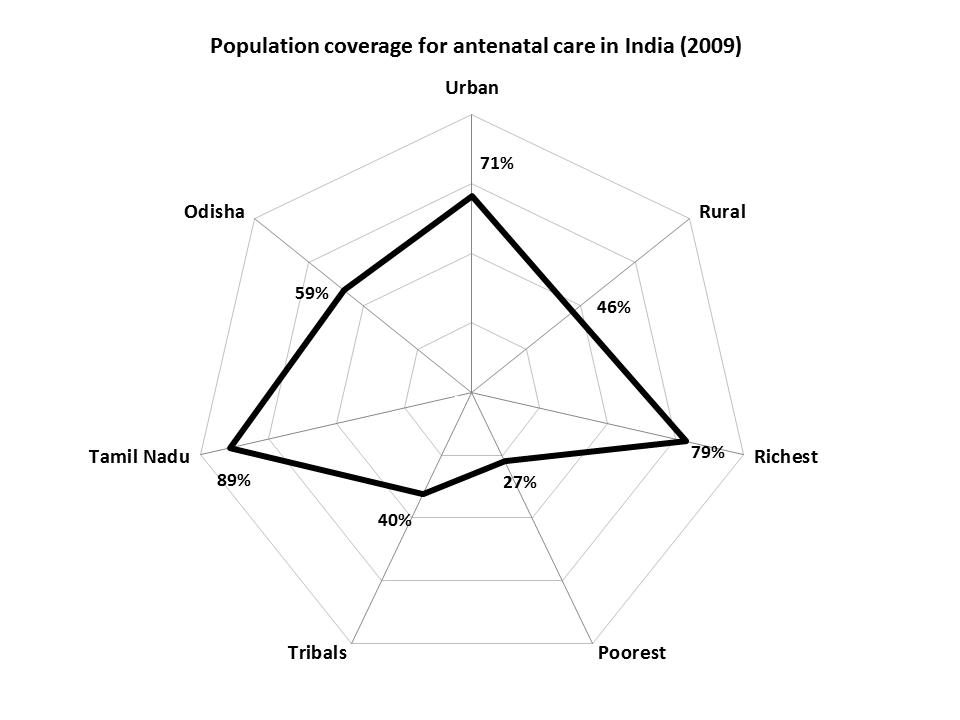


**Figure S4:** Population coverage of antenatal services by different groups in India (2009)

Data Source: Coverage Evaluation Survey (2009)

We used the NSS 60th round data to capture the financial coverage of antenatal check-up and immunisation of children (Figure S5). We note that while 73% of women have received at least one antenatal check-up, only 50% of all pregnant women have received this check-up free of charge. The rest had to pay an average of INR 499 to INR 905 to receive this care, even though antenatal check-up is purportedly free in government health facilities. In the case of immunisation, matters are better as nearly 98% of children have received at least one vaccine and 84% of these children have received it free of charge and did not have to pay any money [24].


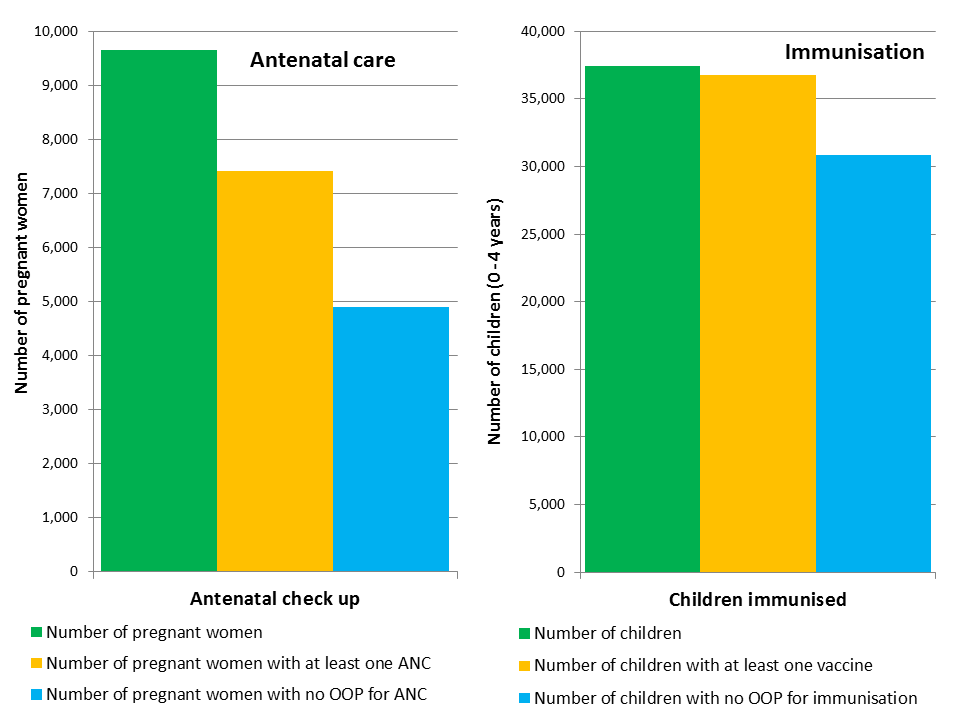


**Figure S5:** Financial coverage for antenatal and immunisation services in India (2006)

Data Source: NSSO 60th round (2006)

Curative care

Curative care is an essential component of any health care package. We looked at natal care, ambulatory care for treatment of acute respiratory ailments in children and outpatient care for rural patients to assess the coverage for curative care. For these three services, we have data on the number of people requiring care as well as the number of people who sought care in a facility; for the first two up to 2009, and for the third up to 2004. Figure S6 clearly shows that the coverage for natal care has improved over time, and there is a modest increase in utilisation of ambulatory care for acute respiratory ailments, but utilisation of outpatient care for rural patients has remained stagnant. Among those women who did not deliver at a facility, the main reasons for not doing so were costs (18%), poor quality (23%) and distance (10%). Similarly, 5% of all rural patients did not seek outpatient treatment quoting financial barriers as the main reason.


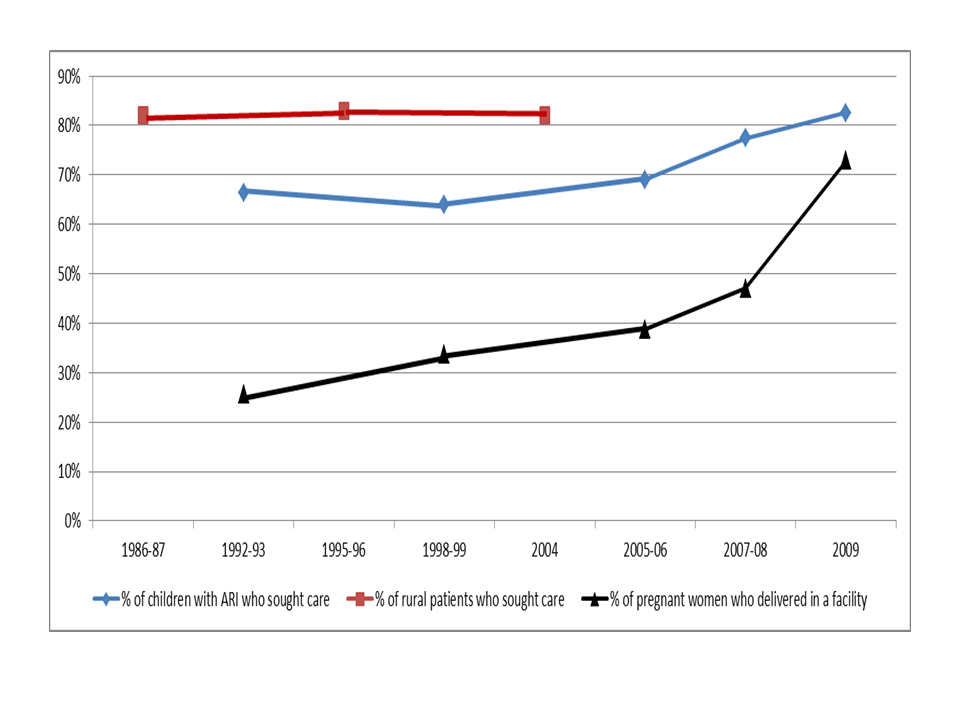


**Figure S6:** Coverage of select curative care services in India (1986 to 2009)

Data Source: NSSO surveys; Coverage evaluation survey and National Family Health Survey

Coverage by select socio-economic-geographic groups also indicates good population coverage for ambulatory and natal care. Even poor families sought out care in facilities for their conditions (Table S3).

|  | Ambulatory care for children with ARI (a) | Natal care (a) | Outpatient care for patients (b) |
| --- | --- | --- | --- |
| Urban | 91% | 86% | 89% |
| Rural | 80% | 68% | 82% |
| Richest quintile | 96% | 90% | 89% |
| Poorest quintile | 71% | 55% | 79% |
| Tribals | 75% | 57% | 75% |
| Tamil Nadu | 89% | 98% | 81% |
| Odisha | 79% | 76% | 77% |

**Table S3:** Population coverage of curative services by different groups in India (2006 / 2009)

Data Sources: (a) – Coverage evaluation survey (2009), (b) – NSSO 60^th^ round (2006)

Of all the patients who were ill in the last 15 days, 85% of them sought outpatient care in a facility and is a reflection of the met need for outpatient services. However, we find that 58% of households with outpatients experienced CHE while seeking care (Figure S1). Similarly, of all the pregnant women who delivered in 2004, only 55% had delivered in an institution. Of these, 58% (or 32% of all women who delivered) experienced CHE due to medical expenses at the time of delivery.

**
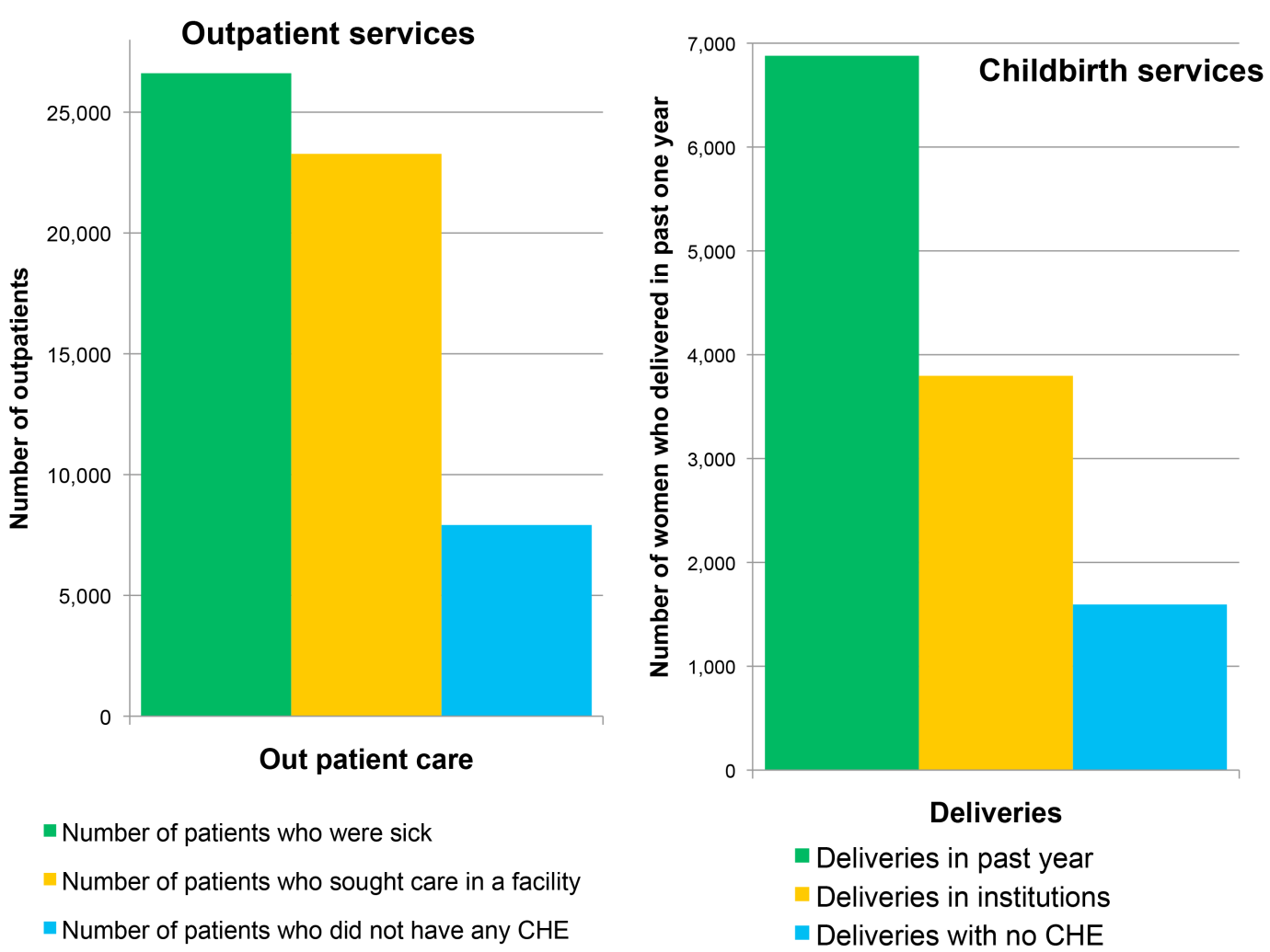
**

**Figure S1**: Financial coverage for outpatient and childbirth services in India (2006).

Data Source: NSS 60th round. 2006.

Of all the households with an inpatient in the past one year (27,617), nearly 61% of the households experienced CHE due to medical expenses at the point of care. One of the main reasons for this is that most of the patients go to private hospitals and incur huge OOP payments, an average of INR 5,695. Unfortunately, patients incur OOP payments, even in the ‘free’ rural government hospitals (an average of US$ 72).

Promotive care

Promotive care addresses some of the social determinants of health, like hygiene and sanitation, diet and lifestyles. We use census data as well as tobacco surveys to document the extent to which Indians are covered by promotive care (Figure S7).


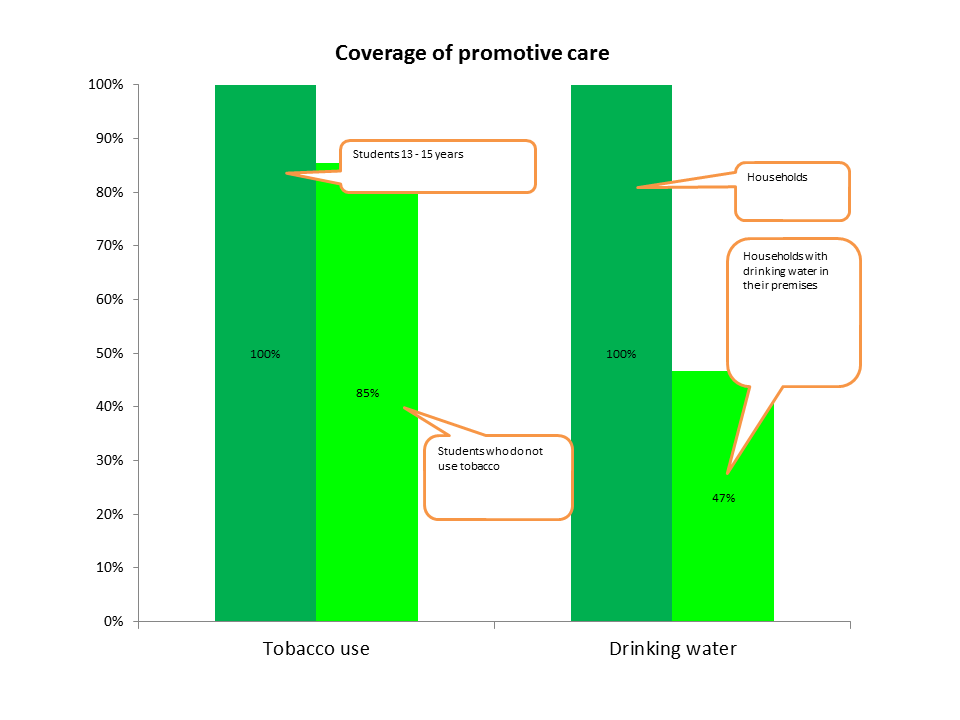


**Figure S7:** Population coverage for promotive care in India (2011)

Data Source: Global Adult Tobacco Survey 2011, Census of India 2011

The Global Adult Tobacco Survey (GATS) interviewed school children and enquired about their awareness and use of tobacco. Approximately a quarter of the students were exposed to tobacco use in their homes and 14% of students were already using tobacco [49].

Safe drinking water is yet another important intervention to promote health. Less than half the population have access to water within their premises. Others have to walk a distance ranging from 100 metres to kilometres to collect water. This situation is worse in rural areas, where the families protected against water borne diseases are only 35%. Even in urban areas, households had to purchase water from private sources. Independent surveys in Mumbai and Chennai indicate that households, especially the poor, spend an average of 5% of their income on water [50, 51].

Figure S2 shows the above information in a single graph, using a modified version of WHO’s UHC cube. From the above figure, it appears that the service coverage was quite reasonable for most services, except for antenatal check-ups, institutional deliveries and access to safe drinking water. The NRHM may have rectified the first two. However, as expected, most services and especially curative care lacked financial coverage. Unfortunately, one does not have any recent or nationally representative data regarding the effect of NRHM and the government sponsored health insurance schemes on OOP payments. A study in Gujarat showed that OOP payments have not reduced for RSBY patients [52]. Another study on the conditional cash transfer for promoting institutional deliveries has suggested that the scheme has increased OOP payments made by the pregnant women [53].

**
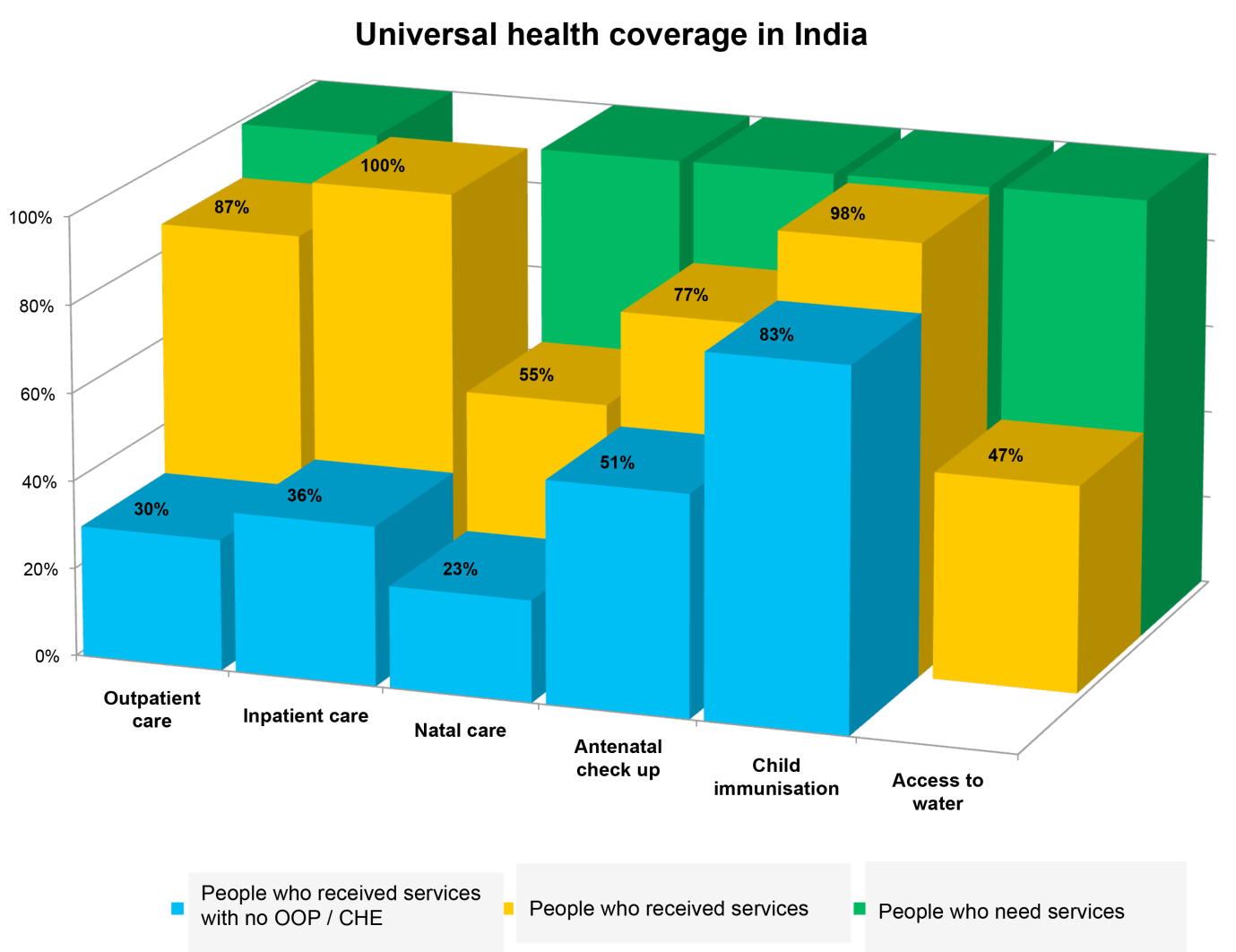
**

**Figure S2:** Universal health coverage for selected health services in India in 2004.

Data Source: Created by the authors, based on previous figures.

**5. Conclusions and recommendations**

The above figures show the extent to which India is on the path to UHC. Due to the presence of both public and private health sector, most of the required health services are available, though they need not be accessible or affordable. Services from primary to tertiary and even rehabilitative services are available for those who can afford to pay [54]. Thus in a sense, India has good service coverage. However, the quality of these services is variable; studies consistently show that the technical quality is low in both the private and government health facilities [55, 56].

Despite decades of emphasis on maternal and child health services by the public health sector [57], only about 60 to 70% of Indians are covered by these preventive services. One positive aspect is that there is reasonable financial coverage in preventive care. Similarly, while most Indians have good access to curative services and are able to utilise the same, most of them also have to make OOP payments at the point of use. Thus while curative care has good service coverage, people are not protected financially when they access curative services. Another issue is the inequity in coverage, especially for preventive services. Poor and aboriginal households do not have access to many of the essential preventive services like antenatal check-ups, leave alone screening for cancer of the cervix.

One of the main reasons for this low coverage is the fact that patients have to make OOP payments when accessing care, even in government hospitals [24]. While preventive care is relatively free, curative care is very expensive for the patient as nearly all of them have to pay to receive the necessary care. If India has to move towards UHC, one of the immediate steps that it needs to take is to reduce this OOP payment, especially for curative care. They would need to ensure that government primary health centres and hospitals are providing the required services and that these services are available free at the point of use. User fees in government hospitals need to be removed and patients should receive not just a consultation but also free medicines and diagnostics. To achieve this, the government needs to spend more money on health care. Many middle income countries spend an average of I$ 250 to I$ 500 per citizen on health care while India spends only I$ 36 [58]. The government needs to spend much more if we want to move towards UHC.

WHO describes financial coverage as access to affordable care [3]. The challenge is to define ‘affordable care’. Some authors have stated that if a household experiences CHE while accessing care, then that care is not affordable [59]. While many have suggested the incidence of CHE as an indicator, it is not clear which definition of CHE to use. Some have defined CHE as total health expenditure that is more than 10% of annual household income [60,61], while others have defined CHE as total health expenditure that is more than 40% of annual disposable household income [59,62]. Yet others have challenged these arbitrary cut off points, stating that any health expenditure can be catastrophic for a poor family. There is the additional problem of patients not accessing care because of the financial barriers. These patients are not included in the numerator while calculating CHE by traditional measures [63]. Wagstaff concludes that, in the absence of an uniform and robust metric for CHE, researchers should continue using the existing (but flawed) measures [64]. Definition of CHE is a topic that requires more research.

Our study had some limitations; for one, the databases that we used were from different surveys and over different periods of time. This may not give us the most accurate estimates of UHC in India. For example, while we have service and population coverage data from 2009, our financial coverage data is only of 2004. So to extrapolate UHC from these different databases may look questionable. However, given the high resources and long time required to do primary data collection across a large country like India, we felt that our method would give the policy makers a rough measure of the status of UHC in India.

Another limitation of our study is that it is slightly dated, with financial figures from 2004 and service figures from 2009. Since then there have been many changes in the Indian health system, especially with the government providing extra resources and programmes for strengthening the health services.

Unfortunately, there is no recent database that provides information on population coverage and financial coverage. So one is not able to calculate the extent to which Indians are currently covered. It is important to note that in spite of these changes (introduction of many programmes including NRHM & RSBY), total government expenditure on health has remained stagnant, hovering around 1% of GDP in the last couple of decades [30].Therefore, we do not expect any significant reduction in the incidence of OOP.

A third limitation of our study is that we do not have any measure to evaluate the quality of care. Thus while 84% of patients received ambulatory care or 55% of women delivered in a facility, we could not comment on whether the care that they received was appropriate and rational and the patients’ health problem was solved.

Currently, the HMIS and other programme information systems do not capture the required data to measure all three dimensions of UHC. So the only way out is to rely on independent surveys that capture data on population, service and financial coverage. Also these surveys need to be more frequent, maybe once in three years as compared to the current practice of once in 10 years.

To conclude, if India wants to move ahead on the path to UHC, then the government needs to invest a lot more money on healthcare. The increased allocation needs to be targeted on improving the coverage of preventive services, especially for the vulnerable; providing 24 x 7 curative services as well as free medicines and diagnostics at all government facilities and improving the quality of care provided so that patients shift from the private to the free government services. These measures may ensure better population coverage as well as financial protection.

| **Box S4: Recommendations**   - In India, the government needs to increase its spending from the current 1% of GDP - This extra money should be used to protect families from out-of-pocket payments at the time of illness as well as should be used to widen the preventive services among the vulnerable population groups. - The government should also set up a monitoring system to monitor the progress that the country is making towards UHC. |
| --- |

**References**

1. World Health Organization (2000) The World Health Report 2000. Health systems: Improving performance. Geneva: WHO.
2. Carrin G, James C, Evans D (2005) Achieving Universal Health Coverage: Developing the health financing system. Geneva.
3. World Health Organization (2010) The World Health Report: health systems financing: the path to universal coverage. Geneva.
4. WHO (2013) Research for Universal Health Coverage. Geneva: World Health Organisation.
5. Morris C (2013) Universal Health Coverage -"The most powerful concept that public health has to offer: 1–5.
6. WHO (2014) What is universal coverage? Available: http://www.who.int/health_financing/universal_coverage_definition/en/. Accessed 7 April 2014.
7. Tanahashi A (1978) Health service coverage and its evaluation. Bull World Heal Organ 56: 295–303.
8. Savedoff WD, Smith AL (2011) Achieving Universal Health Coverage : Learning from Chile , Japan , Malaysia and Sweden. Washington DC.
9. Barnighausen T, Sauerborn R (2002) One hundred and eighteen years of the German health insurance system: are there any lessons for middle and low income countries? Soc Sci Med 54: 1559–1587.
10. Hanvoravongchai P (2013) Health Financing Reform in Thailand : Toward Universal Coverage under Fiscal Constraints. Washington DC.
11. Moreno-Serra R, Smith PC (2012) Does progress towards universal health coverage improve population health? Lancet 380: 917–923. Available: http://www.ncbi.nlm.nih.gov/pubmed/22959388. Accessed 9 August 2013.
12. Bhore J (1946) Report of the health survey and development committee survey Vol 1. New Delhi: Government of India Press.
13. World Health Organization (2008) The World Health Report 2008: Primary Health Care – Now More Than Ever. Geneva.
14. Cehat (2005) Review of Health Care in India. LV G, Duggal R, Shukla A, editors Mumbai: Centre for Equity into Health & Allied Themes .
15. Amrith SS (2009) Health in India Since Independence. London.
16. Banerji D (1985) Health & Family Planning Services in India: An epidemiological, Socio-cultural and Political Analysis and a Perspective. New Dehli: Disha Banerji (Lok Paksh).
17. Ramalingaswami V, Arole RS, Coyaji BJ, Deodhar NS, Jungalwalla N, et al. (1981) HEALTH FOR ALL - an alternative strategy. New Delhi: ICSSR - ICMR.
18. Ministry of Health and Family Welfare (1983) National Health Policy. New Delhi: Government of India.
19. Ministry of Health and Family Welfare (2002) National Health Policy 2002. New Delhi: Government of India.
20. Ministry of Health and Family Welfare (2005) Report of the National Commission on Macroeconomics and Health. New Delhi: Government of India.
21. International Institute for Population Sciences, ORC Macro (2000) NFHS 2 fact sheet - states. National Family Health Survey (NFHS 2) India 1998-99. Mumbai: IIPS.
22. Ministry of Health and Family Welfare (2005) National Rural Health Mission. New Delhi.
23. Ministry of Health and Family Welfare (2005) National rural health mission to move from selective to comprehensive health care. Press Inf Bur: 1–2.
24. National sample survey organisation (2006) Morbidity, Health care and condition of the aged. New Delhi: Government of India.
25. Berman P, Ahuja R, Bhandari L (2010) The Impoverishing Effect of Healthcare Payments in India: New Methodology and Findings. Econ Polit Wkly 45: 65–71.
26. Devadasan N, Swarup A (2008) Rashtriya Swasthya Bima Yojana: an overview. IRDA J 6: 33 – 36.
27. Ministry of Labour and Employment (2014) Rashtriya Swasthya Bima Yojna.
28. La Forgia G, Nagpal S (2012) Government-Sponsored Health Insurance in India: are you covered? Washington DC: World Bank. Available: http://elibrary.worldbank.org/content/book/9780821396186.
29. Bang A, Chatterjee M, Dasgupta J, Garg A, Jain Y, et al. (2012) High level expert group report on Universal Health Coverage for India. New Delhi.
30. Planning Commission (2012) Health Chapter : 12th Plan. 12th Five Year Plan. New Delhi: Government of India. pp. 1–71.
31. Ministry of Health and Family Welfare (2012) Rural Health Statistics in India 2012. New Delhi.
32. Kotwani A, Ewen M, Dey D, Iyer S, Lakshmi PK, et al. (2007) Prices and availability of common medicines at six sites in India using a standard methodology. Indian J Med Res 125: 645–654. Available: http://www.ncbi.nlm.nih.gov/pubmed/17642500.
33. Kotwani A (2013) Where are we now: assessing the price, availability and affordability of essential medicines in Delhi as India plans free medicine for all. BMC Health Serv Res 13: 285. Available: http://www.pubmedcentral.nih.gov/articlerender.fcgi?artid=3733775&tool=pmcentrez&rendertype=abstract. Accessed 11 April 2014.
34. Damrongplasit K, Melnick G a (2009) Early results from Thailand’s 30 Baht Health Reform: something to smile about. Health Aff 28: 457–466. Available: http://www.ncbi.nlm.nih.gov/pubmed/19336469. Accessed 21 August 2013.
35. Jakab M (2012) Measuring progress towards universal health coverage. Measuring progress towards UHC. Madrid: WHO. pp. 1–14.
36. Giedion U, Alfonso E, Diaz Y (2013) The impact of Universal Coverage Schemes in the Developing World: a review of the existing evidence. Washington DC.
37. National Sample Survey Organisation (1998) Morbidity and Treatment of Ailments: NSS 52 Round. 52nd Round. New Delhi: Government of India.
38. Registrar General of India (2013) Estimated birth rate, death rate and infant mortality rate, 2012. SRS Bull 48: 1–6.
39. Registrar General of India (2011) Special bulletin on maternal mortality in India: 2007-09. New Delhi.
40. International Institute for Population Sciences (1995) Executive Summary. National Family Health Survey (MCH and Family Planning), India 1992-93. Mumbai: IIPS. pp. 24–35.
41. International Institute for Population Sciences (2007) National family health survey: 2005–06. Mumbai: International Institute for Population Sciences.
42. International Institute for Population Sciences (2000) Reproductive and Child Health Project. Mumbai: IIPS.
43. International Institute for Population Sciences (2006) Reproductive and child health: India. New Delhi: Ministry of Health and Family Welfare. doi:10.4135/9781412963855.n618.
44. International Institute for Population Sciences (2010) District Level Household and Facility Survey 2007 - 08. New Delhi: Ministry of Health and Family Welfare.
45. UNICEF (2009) Coverage Evaluation Survey: all India report. New Delhi.
46. Registrar General and Census Commissioner (2011) Houses, Household Amenities and Assets: Drinking water. New Delhi: Government of Chhattisgarh.
47. Lal S (1999) Health management information system in rural area. Indian J Community Med 24: 3–8.
48. Belay T, Mbuya N, Rajan V (2009) Data utilization and evidence based decision making in the health sector. India Heal Beat 2: 1–4.
49. International Institute for Population Sciences (2012) Global adult tobacco survey Fact sheet India: 2009-2010. Mumbai.
50. Shaban A (2008) Water Poverty in Urban India : A Study of Major Cities. Mumbai.
51. Venkatachalam L (2012) Role of “Informal Water Markets” in Urban Water Supply: A Household Survey Based Case Study of Chennai City, India. Chennai.
52. Devadasan N, Seshadri T, Trivedi M, Criel B (2013) Promoting universal financial protection: evidence from the Rashtriya Swasthya Bima Yojana (RSBY) in Gujarat, India. Heal Res Policy Syst 11: 29. Available: http://www.pubmedcentral.nih.gov/articlerender.fcgi?artid=3751687&tool=pmcentrez&rendertype=abstract. Accessed 24 March 2014.
53. National Health Systems Resouce Centre (2010) Evaluation Theory and Evaluation Practice: 1–55.
54. Muraleedharan VR (1999) Characteristics and Structure of the Private Hospital Sector in Urban India: A Study of Madras City.
55. Bhatia J, Cleland J (2004) Health care of female outpatients in south-central India: comparing public and private sector provision. Health Policy Plan 19: 402–409.
56. Das J, Holla A, Das V, Mohanan M, Tabak D, et al. (2012) In urban and rural India, a standardized patient study showed low levels of provider training and huge quality gaps. Health Aff 31: 2774–2784. Available: http://www.pubmedcentral.nih.gov/articlerender.fcgi?artid=3730274&tool=pmcentrez&rendertype=abstract.
57. Ministry of Health & Family Welfare (2011) Annual report 2010-11. New Delhi.
58. World Health Organization (2013) World health statistics 2013. Geneva: World Health Organization.
59. Bonu S, Bhushan I, Rani M, Anderson I (2009) Incidence and correlates of catastrophic maternal health care expenditure in India. Health Policy Plan 24: 445–456.
60. Pradhan M, Prescott N (2002) Social risk management options for medical care in Indonesia. Health Econ 11: 431–446. Available: docs\hi-final\Social risk management Indonesia 60 .
61. Van Doorslaer E, O’Donnell O, Rannan-Eliya R, Samanathan A, Adhikari S, et al. (2006) Effect of payments for health care on poverty estimates in 11 countries in Asia: an analysis of household survey data. Lancet 368: 1357–1361.
62. Xu K, David E, Kei K, Zeramdini R, Jan K, et al. (2003) Household catastrophic health expenditure: a multicountry analysis. Lancet 362: 111–117. Available: http://www.thelancet.com/journal/vol362/iss9378/full/llan.362.9378.original_research.26389.1 .
63. Moreno-Serra R, Millett C, Smith PC (2011) Towards improved measurement of financial protection in health. PLoS Med 8: e1001087. Available: http://www.pubmedcentral.nih.gov/articlerender.fcgi?artid=3167798&tool=pmcentrez&rendertype=abstract. Accessed 8 November 2013.
64. Wagstaff A (2008) Measuring Financial Protection in Health. Washington: World Bank.
